# Safety and efficacy of paromomycin/miltefosine/liposomal amphotericin B combinations for the treatment of post-kala-azar dermal leishmaniasis in Sudan: A phase II, open label, randomized, parallel arm study

**DOI:** 10.1371/journal.pntd.0011780

**Published:** 2023-11-21

**Authors:** Brima Musa Younis, Ahmed Mudawi Musa, Séverine Monnerat, Mohammed Abdelrahim Saeed, Eltahir Awad Gasim Khalil, Anas Elbashir Ahmed, Mujahid Ahmed Ali, Ali Noureldin, Gina Muthoni Ouattara, Godfrey M. Nyakaya, Samuel Teshome, Truphosa Omollo, Michael Ochieng, Thaddaeus Egondi, Mildred Mmbone, Wan-Yu Chu, Thomas P. C. Dorlo, Eduard E. Zijlstra, Monique Wasunna, Jorge Alvar, Fabiana Alves

**Affiliations:** 1 Institute of Endemic Diseases, University of Khartoum, Khartoum, Sudan; 2 Drugs for Neglected Diseases initiative, Geneva, Switzerland; 3 Drugs for Neglected Diseases initiative, Nairobi, Kenya; 4 Department of Pharmacy & Pharmacology, Netherlands Cancer Institute, Amsterdam, The Netherlands; 5 Department of Pharmacy, Uppsala University, Uppsala, Sweden; Institute of Postgraduate Medical Education and Research, INDIA

## Abstract

**Background:**

Treatment for post-kala-azar dermal leishmaniasis (PKDL) in Sudan is currently recommended only for patients with persistent or severe disease, mainly because of the limitations of current therapies, namely toxicity and long hospitalization. We assessed the safety and efficacy of miltefosine combined with paromomycin and liposomal amphotericin B (LAmB) for the treatment of PKDL in Sudan.

**Methodology/principal findings:**

An open-label, phase II, randomized, parallel-arm, non-comparative trial was conducted in patients with persistent (stable or progressive disease for ≥ 6 months) or grade 3 PKDL, aged 6 to ≤ 60 years in Sudan. The median age was 9.0 years (IQR 7.0–10.0y) and 87% of patients were ≤12 years old. Patients were randomly assigned to either daily intra-muscular paromomycin (20mg/kg, 14 days) plus oral miltefosine (allometric dose, 42 days)–PM/MF–or LAmB (total dose of 20mg/kg, administered in four injections in week one) and oral miltefosine (allometric dose, 28 days)–LAmB/MF. The primary endpoint was a definitive cure at 12 months after treatment onset, defined as clinical cure (100% lesion resolution) and no additional PKDL treatment between end of therapy and 12-month follow-up assessment. 104/110 patients completed the trial. Definitive cure at 12 months was achieved in 54/55 (98.2%, 95% CI 90.3–100) and 44/55 (80.0%, 95% CI 70.2–91.9) of patients in the PM/MF and AmB/MF arms, respectively, in the mITT set (all randomized patients receiving at least one dose of treatment; in case of error of treatment allocation, the actual treatment received was used in the analysis). No SAEs or deaths were reported, and most AEs were mild or moderate. At least one adverse drug reaction (ADR) was reported in 13/55 (23.6%) patients in PM/MF arm and 28/55 (50.9%) in LAmB/MF arm, the most frequent being miltefosine-related vomiting and nausea, and LAmB-related hypokalaemia; no ocular or auditory ADRs were reported.

**Conclusions/significance:**

The PM/MF regimen requires shorter hospitalization than the currently recommended 60-90-day treatment, and is safe and highly efficacious, even for patients with moderate and severe PKDL. It can be administered at primary health care facilities, with LAmB/MF as a good alternative. For future VL elimination, we need new, safe oral therapies for all patients with PKDL.

**Trial registration:**

ClinicalTrials.gov NCT03399955, https://clinicaltrials.gov/study/NCT03399955

ClinicalTrials.gov ClinicalTrials.gov

## Author summary

Post-kala-azar dermal leishmaniasis (PKDL) is a skin rash which appears months or years after successful treatment of visceral leishmaniasis (VL) caused by *Leishmania donovani*. It is not life-threatening, and since patients with PKDL are otherwise well and may self-heal, they rarely seek treatment. Sandflies (the parasite vector) become infected with *L*. *donovani* after feeding on patients with PKDL, and so, from a public health perspective, ideally all patients, regardless of severity, would be treated to control VL, even those with mild disease. However, current therapies are toxic and/or require long hospitalization. Therefore, treatment is recommended only for patients with persistent disease or severe PKDL. We conducted a phase II trial in 110 patients with PKDL to assess two antimony-free combination therapies and demonstrated that a paromomycin/miltefosine regimen was suitably safe and highly efficacious even for moderate and severe PKDL, and could be administered at primary health centres. This makes it more patient-friendly, which is important since most people with PKDL are children. A liposomal amphotericin B/miltefosine regimen is a good alternative for implementation in hospitals for a shorter period. To support VL elimination in East Africa, new, safe, oral therapies for all patients with PKDL are still needed, including mild cases and women of reproductive potential unwilling or unable to use contraception.

## Introduction

Post-kala-azar dermal leishmaniasis (PKDL) is characterized by a skin rash presenting months or even years after treatment of visceral leishmaniasis (VL) caused by *Leishmania donovani*. In Africa, PKDL occurs mainly in Sudan and Ethiopia, with very few cases reported in other endemic countries in the region. Historically, 50 to 60% of people with VL in Sudan were reported to develop PKDL, when antimonials were the mainstay of VL treatment. A large proportion of these cases (85%) were reported to resolve spontaneously within 12 months, with the majority showing signs of improvement within 6 months [1,2]. The interval between VL treatment and PKDL onset is 0–12 months in Africa, with most cases presenting within 6 months [1].

More recently, a Phase III clinical trial showed PKDL rates of 20.9% for VL patients in Sudan and Ethiopia treated with the current standard of care, a sodium stibogluconate (SSG) and paromomycin (PM) combination, and 4.4% for VL patients treated with a miltefosine and paromomycin combination, after 6 months of follow-up [3]. The reasons for the development of PKDL are not well understood and may depend on host immune responses to *L*. *donovani* [4] or incomplete or inadequate treatment of VL [5]. Studies in India [6], Bangladesh [7], and Sudan [8] show that patients may develop PKDL after effective VL treatment with any of the currently available drugs.

Despite the disease being a manifestation of *L*. *donovani* infection, it is not a life-threatening condition, and the skin lesions are not painful or itchy, thus patients with PKDL are otherwise well and rarely seek treatment, unless the disease progresses, spreading to large body areas. In Africa, most patients present with papular, nodular, or maculo-papular lesions, with a smaller proportion presenting with micro-nodular or macular PKDL [9]. The clinical presentation is described in three grades of severity, taking into account the distribution and the density of lesions [10]. In Sudan, a fifth of patients seeking treatment present with severe and disfiguring PKDL (i.e., grade 3) [1].

There are no conclusive studies on treatment of PKDL in Africa. Usually, PKDL treatments are longer than those for VL, mainly because lesions may take a long time to heal. Studies with a limited number of patients with PKDL in Sudan showed that liposomal amphotericin B (LAmB, 50 mg/kg total dose) had an efficacy of 83% (10/12 patients) [11] and the combination of SSG with PM for 30–40 days had an efficacy of 88.9% (8/9 patients) [12]. The current recommendation for PKDL treatment in Sudan is SSG/PM combination for 17 days or a 60-90-day regimen of SSG, which is associated with life-threatening toxicity and must be administered parenterally in a hospital setting. Moreover, ensuring the sustainable supply of these drugs can be cumbersome. LAmB is usually used for PKDL relapses and complicated cases at 50 mg/kg total dose, administered over 20 days [13]. Since a large proportion of patients may present with mild PKDL and self-heal, and due to the very long, parenteral, and potentially toxic therapies currently available, the risk/benefit is not favourable for most patients. Only patients with severe or persistent disease that do not self-heal within 6 months are recommended for treatment [14,15].

Studies in India and Bangladesh have demonstrated that phlebotomine sandflies can become infected with *L*. *donovani* after feeding on patients with PKDL [16,17], meaning PKDL lesions are a reservoir of the leishmania parasite and may play an important role in transmission during outbreaks or interepidemic periods [9,18]. In Eastern Africa, treatment is recommended for case management of patients with persistent or severe disease. However, for the majority of patients with mild disease, which will cure spontaneously within a few months, there is a balance to be struck between the benefit of controlling VL by reducing the reservoir of patients with PKDL and the risk of giving prolonged courses of potentially toxic and expensive drugs to otherwise well patients.

A suitably safe treatment that can be administered on an out-patient basis, or that requires only a short hospitalization period, is needed. In addition, data on drug exposure in the skin is needed to guide better choices of therapy for PKDL. LAmB in monotherapy and in combination with MF for short courses have been successful for the treatment of PKDL in the Indian subcontinent [19,20], but shorter combination regimens have not been tried in East Africa. The strategy to combine one parenteral drug (LAmB or PM) for a short period (1–2 weeks) with an oral drug (MF) for a longer duration (4–6 weeks) is to limit the hospitalization period, while extending the treatment at home to achieve appropriate exposure and satisfactory efficacy levels.

MF is orally available and has effectively been used in the treatment of various dermal presentations of leishmaniasis [21,22]. Studies on patients with VL in East Africa showed that children require an allometric dosing regimen for miltefosine to reach appropriate levels of exposure, which is associated with cure [3,23,24], therefore the allometric miltefosine regimen was adopted in this study. PM is affordable and available in Sudan [25], it can be administered by intramuscular injection in primary healthcare settings and is stored at room temperature. Although LAmB has satisfactory cure rates for PKDL in Sudan [11] and India [20,26], it requires a cold chain and skilled health professionals, and so we shortened the treatment duration and hospitalization to 1 week for a total dose of 20 mg/kg.

The primary objective of this study was to assess the safety and efficacy of PM combined with MF and LAmB combined with MF for the treatment of patients with PKDL in Sudan who present the disease progressing for more than 6 months, or with grade 3 lesions. A secondary objective was the assessment of skin and plasma concentrations of PM, LAmB, and MF. In addition, an exploratory analysis was done to identify potential risk factors associated with the treatment outcome. This strategy seeks to reduce the hospitalization period, extend the oral treatment to achieve appropriate exposure and satisfactory efficacy levels, and reduce the risk of resistance development. In addition, parasite dynamics in skin and blood were assessed by qPCR, and immunological markers were investigated before, during, and after treatment. These are outside the scope of the present manuscript and will be presented in another article.

## Methods

### Ethics statement

Approval was obtained from the independent ethics committee at the Faculty of Medicine, University of Khartoum and the Sudanese National Medicines and Poisons Board. The study was conducted in accordance with the Declaration of Helsinki, with the International Council for Harmonization Good Clinical Practice (GCP) guidelines, and following all applicable state, local, and foreign laws for protecting the rights and welfare of human subjects. Informed consent and assent (when applicable) were obtained as per regulatory requirements. Written voluntary informed consent was obtained from adult patients and from the parent or guardian of any child <18 years old; assent from minors was also obtained according to country regulations.

### Study design and participants

This open-label, phase II, randomized, parallel-arm, non-comparative trial was conducted at the Professor El-Hassan Centre for Tropical Medicine, Doka, Sudan. Patients were randomized to one of two arms: PM for 14 days with MF for 42 days (arm 1) or LAmB for 7 days with MF for 28 days (arm 2). Patients aged 6 to ≤ 60 years with documented stable or progressive disease for at least 6 months or grade 3 PKDL, confirmed by clinical presentation and demonstration of parasites in a skin smear by microscopy or polymerase chain reaction (PCR), were included. Women of childbearing potential were eligible to participate but were required to use contraception from the beginning of the treatment period until 5 months after the end of treatment. Exclusion criteria included receiving treatment for PKDL within the last year; being pregnant or lactating; signs and symptoms of severe disease (concomitant severe infection or any other serious known underlying disease); severe malnutrition; haemoglobin <5 g/dL; other skin diseases; abnormal liver function (alanine aminotransferase and aspartate aminotransferase tests >3 x normal range); total bilirubin levels >1.5 times the upper normal range; serum creatinine above the upper limit of normal range; serum potassium <3.5 mmol/L; pre-existing clinical hearing loss based on audiometry at baseline; history of allergy or hypersensitivity to one of the study drugs; and being on immunomodulatory therapy. Data on the sex of participants was collected based on self-report. No difference in treatment efficacy was expected between male and female patients.

### Procedures

Arm 1 comprised a once-daily intramuscular injection of 20 mg/kg/day PM (Gland Pharma Ltd.) for 14 days and oral MF (Impavido) BID for 42 days. Arm 2 comprised 5 mg/kg/day intravenous infusion of LAmB on days 1, 3, 5, and 7 (at a total dose of 20 mg/kg) and oral MF BID for 28 days. Children ≤30kg received MF allometric dosing based on sex, weight, and height [3,27]; patients >30kg to <45kg received 100mg/day, and patients ≥45kg received 150mg/day; doses were administered BID with food. Patients were hospitalized for 14 days until the end of PM treatment in arm 1 and for 7 days until the end of LAmB treatment in arm 2. In both arms, MF treatment was started at the same time as PM or LAmB treatment and continued on an out-patient basis after patients were discharged from the hospital, until treatment completion.

Rescue treatment (2.5 mg/kg for 20 days LAmB or, in case of poor tolerance to LAmB, rescue treatment was given at the discretion of the study physician/ investigator) was indicated in case of: failure to respond to treatment at the end of treatment (EOT, Day 42) or 3 months follow-up, progression of disease (lesions worsening over time), reappearance/ reactivation of lesions after an initial healing period, or unable to tolerate the study medication.

For arms 1 and 2, in-patient administration of MF was directly observed; adherence was expected to be 100%, unless the patient vomited and could not tolerate re-administration within 30 minutes. PM and LAmB were administered by study staff. Adherence to outpatient treatment of MF was assessed by collecting the drug packaging and checking for any unused drug, and using a simple diary. Outpatient MF treatment was under direct observed treatment (DOT) by the community health worker at the village level, whenever possible, on a daily basis, or followed using telephone contact until completion of MF treatment. Full compliance was assumed if 90–110% of the prescribed doses were administered.

Samples of blood and skin for PK measurements of MF, PM, and LAmB were taken according to the schedule of assessments presented in Table A in S1 File. Skin biopsies (2 mm) were taken with a sterile disposable biopsy punch from the active border of a skin lesion. Blood samples were collected in ethylenediaminetetraacetic acid (EDTA) tubes. Samples were analyzed using liquid chromatography coupled to tandem mass spectrometry assays validated according to the United States Food and Drug Administration and European Medicines Agency guidelines. The development and validation of the MF plasma assay has previously been published [23], while the MF skin assay was developed and validated during this study [28]. The PM and LAmB assays were validated during the preparation of the study [29,30].

### Assessments

Physical examination and vital signs, haematological, biochemical, and safety assessments were carried out in the screening period (day -30 to day 0) and on days 1, 3, 7, 14, and 28. A first clinical assessment for efficacy was made at D42 (+2 days). All patients had follow-up visits at 3 months (±7 days), 6 months (±7 days), and 12 months (±14 days) after the onset of treatment, to assess safety and efficacy. Plasma samples for PK analysis were collected on Days 1, 7, 14, 28, and 42, and at 3 months (sparse and intensive sampling). Skin biopsies (2 mm) were taken for PK analysis on Days 7 and 28 for patients in arm 2, or Days 14 and 42 for patients in arm 1. See Table A in S1 File for the schedule of assessments.

PKDL diagnosis was confirmed in a skin slit smear by direct microscopy (material from the PKDL lesion was smeared on glass slides, air dried, fixed in methanol, stained with Giemsa, and examined microscopically using a 100× oil immersion lens for visualization of the amastigote form of the leishmania parasite) or by PCR using a validated method [31,32].

Clinical evolution was systematically recorded using the PKDL grading to assess disease severity by lesion distribution and density (see Text A in S1 File) [33] and a scoring system developed and validated by Mondal et al. [16]. For lesion distribution grading, grade 1 = mainly on the face with some lesions on the trunk and arms, grade 2 = face, upper parts of the trunk, arms, and legs (gradually becoming less distal), hands and feet are free of lesions, and grade 3 = all over the body, including the hands and feet. For lesion density, grade 1 = scattered lesions, grade 2 = moderate density with normal skin in between lesions, grade 3 = dense rash, no normal skin [26][15]. Clinical assessment to characterize the PKDL lesions was done during the screening period (Days -30 to 0), at day 42 (end of treatment), and at the 3, 6, and 12-month follow-up visits. Using the scoring system described by Mondal et al, skin lesions were plotted in squares corresponding to the affected skin areas of the body [34]. The total number of squares containing lesions were counted at screening, at end of treatment (Day 42), and at 3-, 6-, and 12-months follow-up. % skin lesions cured = number of squares affected at screening ‐ number of squares affected after treatment x 100 / total number of squares affected at screening.

### Endpoints

The primary efficacy endpoint was definitive cure at 12 months after treatment onset, defined as clinical cure (100% lesion resolution) and no additional PKDL treatment between end of therapy and 12-month follow-up assessment. Safety endpoints were serious adverse events (SAEs) from the start of treatment to 12 months follow-up, frequency and severity of adverse events (AEs) that lead to treatment discontinuation, and frequency and severity of all AEs from the start of treatment through 12 months follow-up. A secondary pharmacokinetic endpoint was to assess the maximum drug concentration in the blood plasma at the end of treatment (Day 42) and the maximal accumulation of PM, total amphotericin B, and MF in the skin at the end of treatment (Day 42), and to correlate these values.

### Statistical analysis

A minimum of 50 patients per treatment arm was estimated to provide a precision estimate of 10% with 95% CI, based on an anticipated cure rate of 85% at 12 months. Five patients were added to allow for 10% loss of patients during follow-up, resulting in a sample size of 55 patients per treatment arm and an overall sample size of 110. This was a non-comparative trial.

A computer-generated randomization code was used for patient treatment allocation to one of the two treatment arms in a 1:1 ratio. A set of individual opaque, sealed, and sequentially numbered envelopes was provided to the treatment sites, with one envelope per patient containing the identity of the treatment.

The primary efficacy analysis was performed on the modified intention-to-treat (mITT, all randomized patients receiving at least one dose of treatment; in case of erroneous treatment allocation, the actual treatment received was to be used in the analysis) and per protocol (PP, patients in the mITT with no major protocol deviations) population sets. Missing efficacy outcome at 12 months was considered as treatment failure. A sensitivity analysis of the primary efficacy analysis was performed using the sets of completers (subsets of patients belonging to the mITT or PP who had an outcome at 12 months follow-up i.e., patients with missing efficacy outcome at 12 months were excluded). The primary efficacy endpoint was reported as the proportion of cured patients with a corresponding 95% confidence interval (CI). No interim efficacy analysis was planned; a data safety monitoring board monitored the study to ensure the safety of the participants.

Skin lesion score, distribution grade, and density grade were presented graphically, and Kaplan-Meier survival curves were produced to demonstrate the time to treatment failure and the time to complete cure by treatment arm and for the overall population.

Univariate logistic regression was used to assess the association of baseline PKDL grade, initial disease status, duration of PKDL onset, age, and sex with the risk of failure. The results were presented as odds ratios with the corresponding 95% confidence interval.

The safety analysis was performed on the mITT set, based on treatment-emergent adverse events (TEAEs). The numbers and percentages of patients presenting TEAEs were summarized by treatment arm and overall. All AEs were categorized by system organ class (SOC) and preferred term (PT) according to the Medical Dictionary for Regulatory Activities (MedDRA) Version 24.0, severity (based on the CTCAE v4.03), seriousness and by causal relationship to study drugs. This trial is registered at ClinicalTrials.gov, NCT03399955.

For the pharmacokinetic analysis, a skin to plasma concentration ratio was calculated for each individual for whom a paired skin drug concentration and plasma drug concentration (measured on the same end of treatment day) was available.

## Results

### Participants

**Fig 1:**
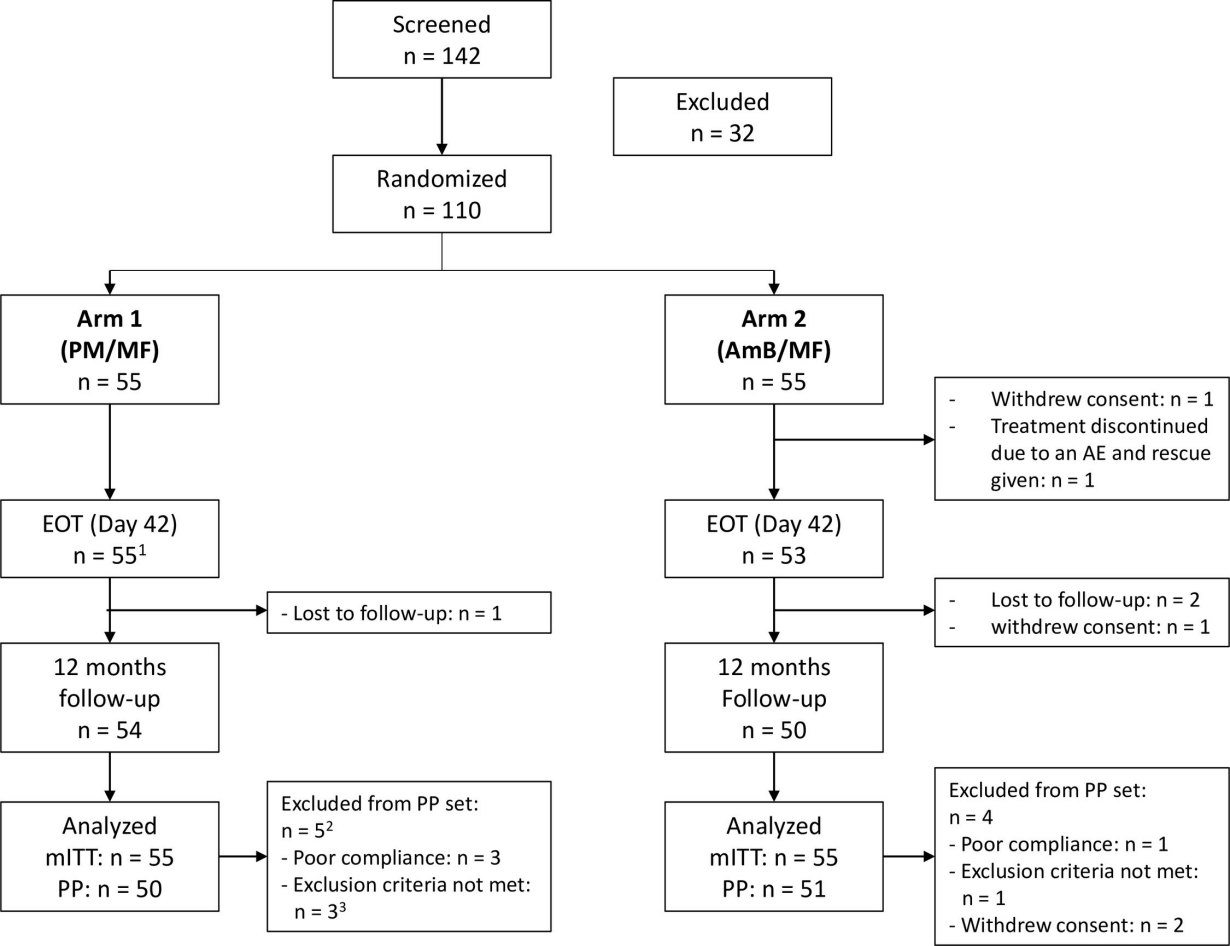
**Patient disposition**. AE = Adverse event; AmB = liposomal amphotericin B; EOT = End of treatment; mITT = Modified intention-to-treat; MF = Miltefosine; PM = Paromomycin; PP = Per-protocol. ^1^ One patient discontinued the study treatment due to an AE but did not receive rescue treatment and completed the study. ^2^ One patient had two major protocol deviations (exclusion criteria met and poor compliance). ^3^ Serum potassium <3.5 mmol/L, but within the normal laboratory range.

The study was carried out between 10th May 2018 and 2nd May 2021. 142 patients were screened and 110 were randomly assigned to one of the two treatment arms: PM/MF (n = 55, 21 female and 34 male) and LAmB/MF (n = 55, 20 female and 35 male). 108/110 (98%) patients reached EOT and 104/110 (95%) reached the end of the study. One male patient did not complete treatment due to withdrawal of consent during the treatment period and another male patient due intolerance to the study treatment, requiring rescue therapy (both in arm 2). Three male patients were lost to follow-up (one in arm 1, two in arm 2) and one female patient withdrew consent during the follow-up period (arm 2) see patient disposition (Fig 1).

Major protocol deviations were recorded in 5 patients in arm 1 (including one patient with two major protocol deviations: 3 did not meet eligibility criteria and 3 had poor compliance to treatment (>±10%)) and 4 patients in arm 2 (1 did not meet eligibility criteria, 1 had poor compliance to treatment (>±10%), and 2 withdrew consent). All patients were included in the mITT set and 101/110 (92%) of patients in the PP set. 105/110 (95.5%) and 98/110 (89.1%) patients were included in the completers set for the mITT and PP sets, respectively.

Demographic and baseline characteristics were comparable between treatment arms. The median age was 9.0 years (IQR 7.0–10.0y) and 87% of patients were ≤12 years old. There was a higher proportion of male patients (62.7%), consistent with the overall VL population in the region. All patients had a previous medical diagnosis of VL, with very few patients having a previous diagnosis of PKDL (8/110, 7.3%). Time from onset of PKDL symptoms to baseline visit ranged from 6 months to 12 years, with a median time of 18 months (IQR 11–34); 7 patients had been previously treated for PKDL more than one year prior to enrolment. 82/110 (74.5%) patients had stable, 28/110 (25.5%) had worsening, and no patients had improving PKDL at baseline. 85/110 (77.3%) patients presented with maculopapular lesions, 20/110 (18.2%) with papular lesions, 3/110 (2.7%) with macular, and 2/110 (1.8%) with plaque-like lesions. Mean skin lesion scores were similar between treatment arms, with an overall mean score of 50.9 (SD 67.0) and a median of 23.0 (IQR 11, 57). The majority of patients presented with grade 1 PKDL lesions by both distribution (84/110 [76.4%]) and density (71/110 [64.5%]). 26/110 patients (23.6%) had grade 2 or 3 PKDL by distribution and 39/110 (35.5%) had grade 2 or 3 PKDL by lesion density (see Table 1 for detailed information per treatment arm). Baseline laboratory parameters were also similar between treatment arms and mostly within the range expected for patients with PKDL (see Table B in S1 File). Compliance to all treatments was >96% during hospitalization, and 92.7% and 98.1% (to MF) during home treatment in arms 1 and 2, respectively.

**Table 1:** Baseline characteristics by treatment arm and overall–ITT set.

Parameter	Statistics	Arm 1 PM/MF	Arm 2 LAmB/MF	Overall
		(n = 55)	(n = 55)	(n = 110)
	Range (Min; Max)	6.0; 30.0	6.0; 19.0	6.0; 30.0
Age (years)	Mean (SD)	9.8 (4.8)	9.2 (2.9)	9.5 (4.0)
	Median (IQR)	9.0 (7; 11)	9.0 (7; 10)	9.0 (7; 10)
Sex	Female, n (%)	21 (38.2)	20 (36.4)	41 (37.3)
	Male, n (%)	34 (61.8)	35 (63.6)	69 (62.7)
Weight (kg)	Range (Min; Max)	15.0; 74.0	15.0; 51.0	15.0; 74.0
	Mean (SD)	26.9 (12.3)	25.3 (8.8)	26.1 (10.7)
	Median (IQR)	24.0 (19.0; 30.0)	23.0 (18.0; 30.0)	23.6 (19.0; 30.0)
Height (cm)	Range (Min; Max)	98.0–186.0	100.0–173.0	98.0–186.0
	Mean (SD)	130.3 (20.2)	128.5 (16.2)	129.4 (18.2)
	Median (IQR)	127.0 (114.0; 143.0)	128.0 (115.0; 142.0)	128.0 (115.0; 142.0)
Previous PKDL diagnosis	Yes, n (%)	3 (5.5)	5 (9.1)	8 (7.3)
	No, n (%)	52 (94.5)	50 (90.9)	102 (92.7)
Previous PKDL treatment^1^	Yes, n (%)	3 (100.0)	4 (80.0)	7 (87.5)
	No, n (%)	0 (0.0)	1 (20.0)	1 (12.5)
Time since onset of PKDL symptoms (months)	Range (Min; Max)	6.5; 152.5	6.7; 70.2	6.5; 152.5
	Mean (SD)	30.9 (33.4)	23.3 (15.5)	27.1 (26.2)
	Median (IQR)	18.5 (9; 40)	20.4 (11; 29)	18.6 (11; 34)
Condition of the PKDL	Stable, n (%)	43 (78.2)	39 (70.9)	82 (74.5)
	Improving, n (%)	0 (0.0)	0 (0.0)	0 (0.0)
	Worsening, n (%)	12 (21.8)	16 (29.1)	28 (25.5)
PKDL type	Macular, n (%)	1 (1.8)	2 (3.6)	3 (2.7)
	Maculopapular, n (%)	41 (74.5)	44 (80.0)	85 (77.3)
	Papular, n (%)	11 (20.0)	9 (16.4)	20 (18.2)
	Plaque-like, n (%)	2 (3.6)	0 (0.0)	2 (1.8)
Skin lesion score	Range (Min; Max)	6.0; 289.0	7.0; 350.0	6.0; 350.0
	Mean (SD)	48.2 (65.8)	53.7 (68.6)	50.9 (67.0)
	Median (IQR)	22.0 (10; 47)	24.0 (13; 62)	23.0 (11; 57)
PKDL distribution grade	Grade 1, n (%)	43 (78.2)	41 (74.5)	84 (76.4)
	Grade 2, n (%)	9 (16.4)	11 (20.0)	20 (18.2)
	Grade 3, n (%)	3 (5.5)	3 (5.5)	6 (5.5)
PKDL density grade	Grade 1, n (%)	35 (63.6)	36 (65.5)	71 (64.5)
	Grade 2, n (%)	18 (32.7)	15 (27.3)	33 (30.0)
	Grade 3, n (%)	2 (3.6)	4 (7.3)	6 (5.5)

LAmB = Liposomal amphotericin B; IQR = Interquartile range; MF = Miltefosine; ITT = Intention-to-treat; PM = Paromomycin; SD = Standard deviation. Max = Maximum; Min = Minimum; PKDL = Post-kala-azar dermal leishmaniasis; VL = Visceral leishmaniasis.

^1^The percentage of patients with previous PKDL treatment is based on the number (n) of patients previously diagnosed with PKDL. Note: total percentages may not add up to 100.0% due to rounding of percentages in each category.

### Efficacy

Definitive cure at 12 months follow-up was achieved in 54/55 (98.2%) patients in arm 1 and 44/55 (80.0%) in arm 2 in the mITT set. Similar results were observed in the PP set (Table 2). The sensitivity analysis in the set of completers (excluding any missing 12-month efficacy outcomes) confirmed these results with 54/54 (100.0%) patients achieving definitive cure at 12 months in arm 1 and 44/51 (86.3%) in arm 2 in the mITT set (see Table C in S1 File).

**Table 2:** Primary efficacy outcome of definitive cure at 12 months by treatment arm–mITT and PP sets.

	mITT		PP	
Statistics	Arm 1 PM/MF	Arm 2 LAmB/MF	Arm 1 PM/MF	Arm 2 LAmB/MF
n	55	55	50	51
Number cured	54	44	49	42
Efficacy, %	98.2	80.0	98.0	82.4
95% CI	(90.3; 100.0)	(70.2; 91.9)	(89.4; 99.9)	(69.1; 91.6)

LAmB = Liposomal amphotericin B; CI = Confidence interval; MF = Miltefosine; mITT = Modified intention-to-treat; PM = Paromomycin; PP = Per-protocol. In this primary analysis, missing efficacy outcome at 12 months are defined as treatment failure.

Treatment failure was reported in 7 patients in arm 2, all of whom received rescue treatment: 5 due to relapse at 12 months follow-up, 1 absence of cure, and 1 patient with an AE of hypersensitivity related to LAmB who received rescue treatment after treatment discontinuation. Four remaining patients were assigned as failure in the mITT analysis in arm 2 due to withdrawal of consent during treatment (1 patient), withdrawal of consent during follow-up (1 patient) and loss to follow-up (2 patients). In arm 1 the only case of failure in the mITT analysis was 1 patient lost to follow-up.

**Fig 2:**
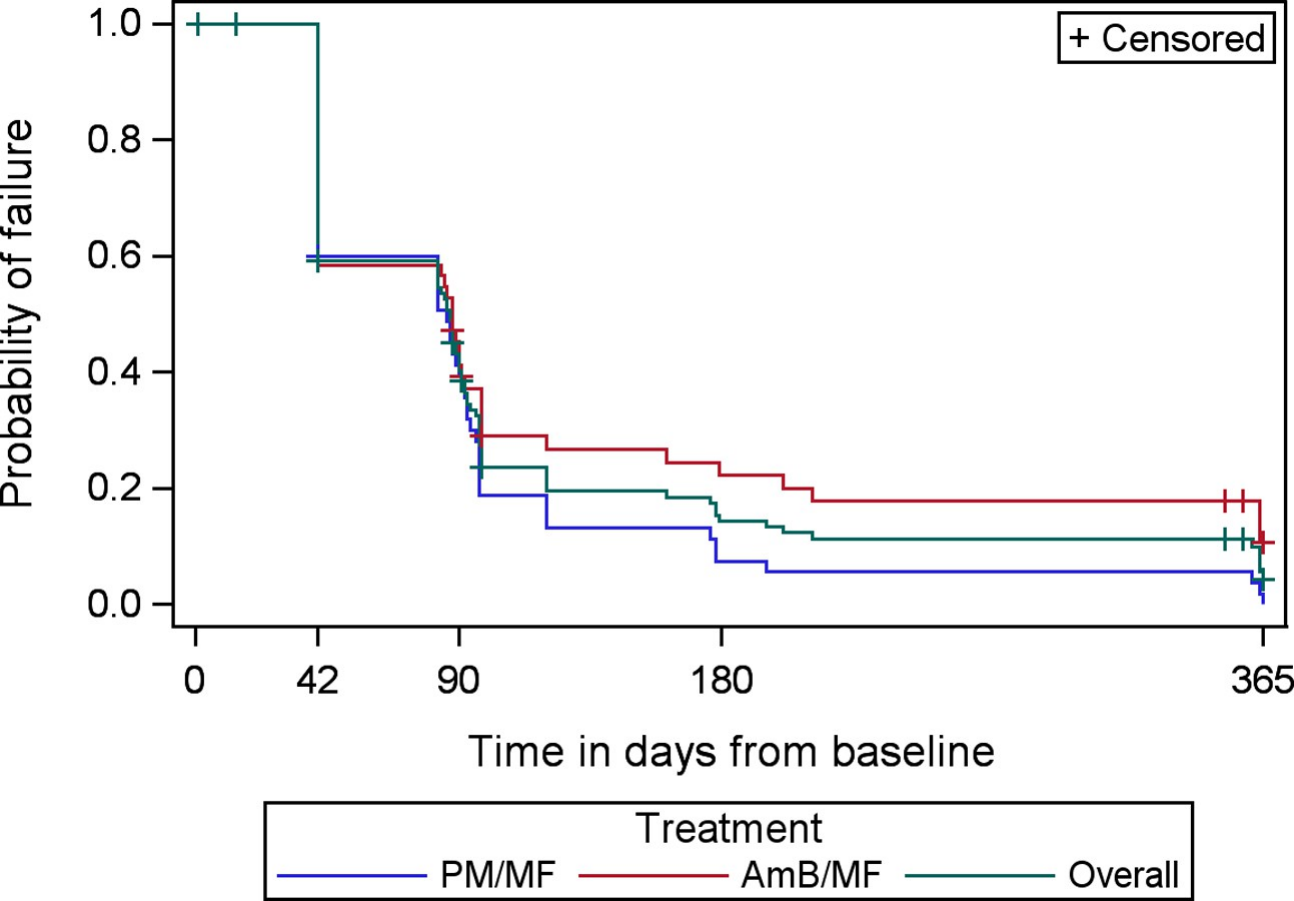
Kaplan-Meier curve of time to complete cure by treatment arm and overall–mITT set.

**Fig 3:**
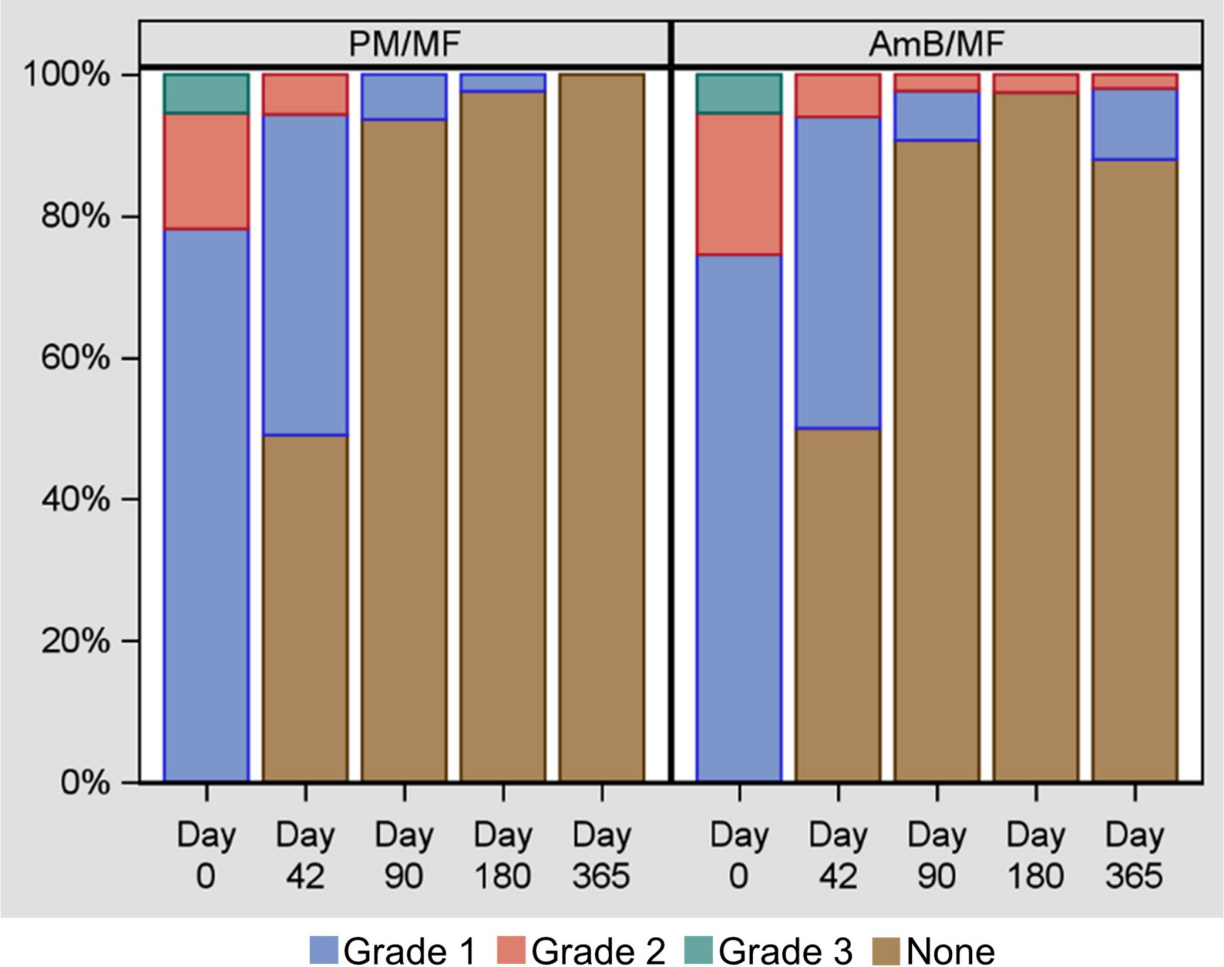
PKDL lesion distribution grade over time by treatment arm–mITT set. AmB = liposomal amphotericin B; MF = Miltefosine; mITT = Modified intention-to-treat; PKDL = Post-kala-azar dermal leishmaniasis; PM = Paromomycin. Note: The grade “none” refers to the absence of lesions in the patients, i.e., the patients are cured.

A Kaplan-Meier analysis of the time to complete cure is presented in the mITT set by treatment arm and overall in Fig 2 and the PKDL lesion distribution grade over time is given in Fig 3. Details of the skin lesion score and lesion density grade over time by treatment arm are given in Figs A and B in S1 File. Nearly half of patients had achieved complete cure at Day 42(EOT), with overall marked improvement in mean skin lesion score and PKDL lesion distribution and density by EOT. At the 3 months follow-up visit, 75% (83/110) of patients achieved complete cure, 6.4% (7/110) of patients remained with lesions and 20 patients had a missing outcome. One patient in arm 2 improved during follow-up, but never completely healed, and lesions worsened by the end of the study, and 5 other patients in arm 2 showed reactivation of lesions at 12-months, at the end of the study, while no relapses were observed in arm 1 (see Fig C in S1 File). Five of the 6 cases of no response or relapse in arm 2 were patients with PKDL grade 2 or 3 at baseline.

A *post-hoc* analysis of the overall odds of treatment failure demonstrated a statistically significant 10-fold increase in risk of failure in patients with grade 3 PKDL lesions (odds ratio of 10.17 [95% CI: 1.2; 85.7], p = 0.033) when considering all patients in both treatment arms. Worsening disease status at baseline was also identified as possibly increasing the odds of failure (odds ratio of 4.11), but no statistical significance was observed (95% CI: 0.9; 19.7, p = 0.077). No other statistically significant risk factors (duration of PKDL onset, age, or sex) were identified (see Table D in S1 File).

### Safety

At least one TEAE occurred in 61/110 (55.5%) of patients, mostly during the treatment period or within 3 months of follow-up, with 25/55 (45.5%) and 36/55 (65.5%) in arms 1 and 2, respectively (Table 3). Most TEAEs were mild or moderate (CTCAE grade 1 or 2). At least severe TEAEs (CTCAE grade ≥3) were reported in 16 patients (14.5%), 5/55 in arm 1 (9.1%) and 11/55 in arm 2 (20.0%), the most common being hypokalaemia (8.2%) and neutropenia (2.7%). Life-threatening TEAEs (CTCAE grade 4) were reported in 3 patients (2.7%, 2 cases of hypokalemia related to LAmB in arm 2 and one case of neutropenia not related to study drugs in arm 1), but did not have associated clinical manifestations or meet SAE criteria based on the investigator’s clinical evaluation of the patients. At least one adverse drug reaction (ADR, AEs related to study drug) was reported in 41/110 (37.3%) patients, 13/55 (23.6%) in arm 1 and 28/55 (50.9%) in arm 2, the most common being MF-related vomiting (30.9%), MF-related nausea (5.5%), LAmB-related hypokalaemia (6.4%), and PM-related injection site pain (2.7%) (Table 4). The higher frequency of ADR in arm 2 was mostly due to vomiting. A *post-hoc* analysis demonstrated a 3-fold increase in the likelihood of vomiting in arm 2 compared to arm 1 (p = 0.008). Most of the ADRs were mild or moderate; only 8 ADRs were CTCAE grade >3 (14.5%), all of them in arm 2 (hypokalaemia or ALT/AST increase). One patient in each arm discontinued treatment (one case of PM and MF-related acute kidney injury (grade 2) in arm 1 that did not require rescue therapy and one case of drug hypersensitivity (grade 2) to LAmB in arm 2 that was rescued). Audiometric tests remained normal in all patients tested (i.e., all patients allocated to Arm 1 [PM/MF]). One case of conjunctivitis was observed in Arm 2, starting at Day 2, which was considered not related to study drug; topical treatment was given for 11 days, with complete resolution. No SAEs or deaths were reported.

**Table 3:** Summary of treatment emergent adverse events–mITT set.

Description	Arm 1 PM/MF	Arm 2 LAmB/MF	Overall
	(n = 55)	(n = 55)	(n = 110)
**Any TEAE**	**25 (45.5)**	**36 (65.5)**	**61 (55.5)**
Any at least severe TEAE^1^	5 (9.1)	11 (20.0)	16 (14.5)
Any SAE	0 (0.0)	0 (0.0)	0 (0.0)
Any TEAE leading to treatment discontinuation	1 (1.8)	1 (1.8)	2 (1.8)
Any TEAE leading to death	0 (0.0)	0 (0.0)	0 (0.0)
**Any treatment-emergent ADR**	**13 (23.6)**	**28 (50.9)**	**41 (37.3)**
Any at least severe treatment-emergent ADR^1^	0 (0.0)	8 (14.5)	8 (7.3)
Any serious ADR	0 (0.0)	0 (0.0)	0 (0.0)
Any treatment-emergent ADR leading to study treatment discontinuation	1 (1.8)	1 (1.8)	2 (1.8)
Any treatment-emergent ADR leading to death	0 (0.0)	0 (0.0)	0 (0.0)

ADR = Adverse drug reaction; LAmB = Liposomal amphotericin B; CTCAE = Common Terminology Criteria for Adverse Events; MF = Miltefosine; mITT = Modified intention-to-treat; PM = Paromomycin; SAE = Serious adverse event; TEAE = treatment-emergent adverse event. Data are presented as n (%) of patients with at least one event.

^1^Events with CTCAE Grade ≥3, i.e., classified as severe (CTCAE Grade 3) or life-threatening (CTCAE Grade 4). There were no events classified as CTCAE Grade 5 (death).

**Table 4:** Treatment-emergent ADRs by SOC and PT by treatment arm and overall–mITT set.

System Organ Class	Preferred Term	Arm 1 PM/MF	Arm 2 LAmB/MF	Overall
		n = 55	n = 55	n = 110
Any treatment-emergent ADR		13 (23.6) [50]	28 (50.9) [90]	41 (37.3) [140]
Gastrointestinal disorders		12 (21.8) [43]	25 (45.5) [78]	37 (33.6) [121]
	Vomiting	10 (18.2) [40]	24 (43.6) [73]	34 (30.9) [113]
	Nausea	3 (5.5) [3]	3 (5.5) [4]	6 (5.5) [7]
	Abdominal pain	0 (0.0) [0]	1 (1.8) [1]	1 (0.9) [1]
Metabolism and nutrition disorders		0 (0.0) [0]	7 (12.7) [8]	7 (6.4) [8]
	Hypokalemia	0 (0.0) [0]	7 (12.7) [7]	7 (6.4) [7]
	Decreased appetite	0 (0.0) [0]	1 (1.8) [1]	1 (0.9) [1]
General disorders and administration site conditions		3 (5.5) [3]	1 (1.8) [1]	4 (3.6) [4]
	Injection site pain	3 (5.5) [3]	0 (0.0) [0]	3 (2.7) [3]
	Chills	0 (0.0) [0]	1 (1.8) [1]	1 (0.9) [1]
Infections and infestations		3 (5.5) [3]	0 (0.0) [0]	3 (2.7) [3]
	Injection site abscess	2 (3.6) [2]	0 (0.0) [0]	2 (1.8) [2]
	Injection site infection	1 (1.8) [1]	0 (0.0) [0]	1 (0.9) [1]
Immune system disorders		0 (0.0) [0]	1 (1.8) [1]	1 (0.9) [1]
	Drug hypersensitivity	0 (0.0) [0]	1 (1.8) [1]	1 (0.9) [1]
Investigations		0 (0.0) [0]	1 (1.8) [2]	1 (0.9) [2]
	Alanine aminotransferase increased	0 (0.0) [0]	1 (1.8) [1]	1 (0.9) [1]
	Aspartate aminotransferase increased	0 (0.0) [0]	1 (1.8) [1]	1 (0.9) [1]
Renal and urinary disorders		1 (1.8) [1]	0 (0.0) [0]	1 (0.9) [1]
	Acute kidney injury	1 (1.8) [1]	0 (0.0) [0]	1 (0.9) [1]

ADR = Adverse drug reaction; LAmB = Liposomal amphotericin B; MF = Miltefosine; mITT = Modified intention-to-treat; PM = Paromomycin; PT = Preferred term; SOC = System organ class; TEAE = Treatment-emergent adverse event. Data are presented as n (%) of patients with at least one event and number [n] of events.

### Pharmacokinetics

**Fig 4:**
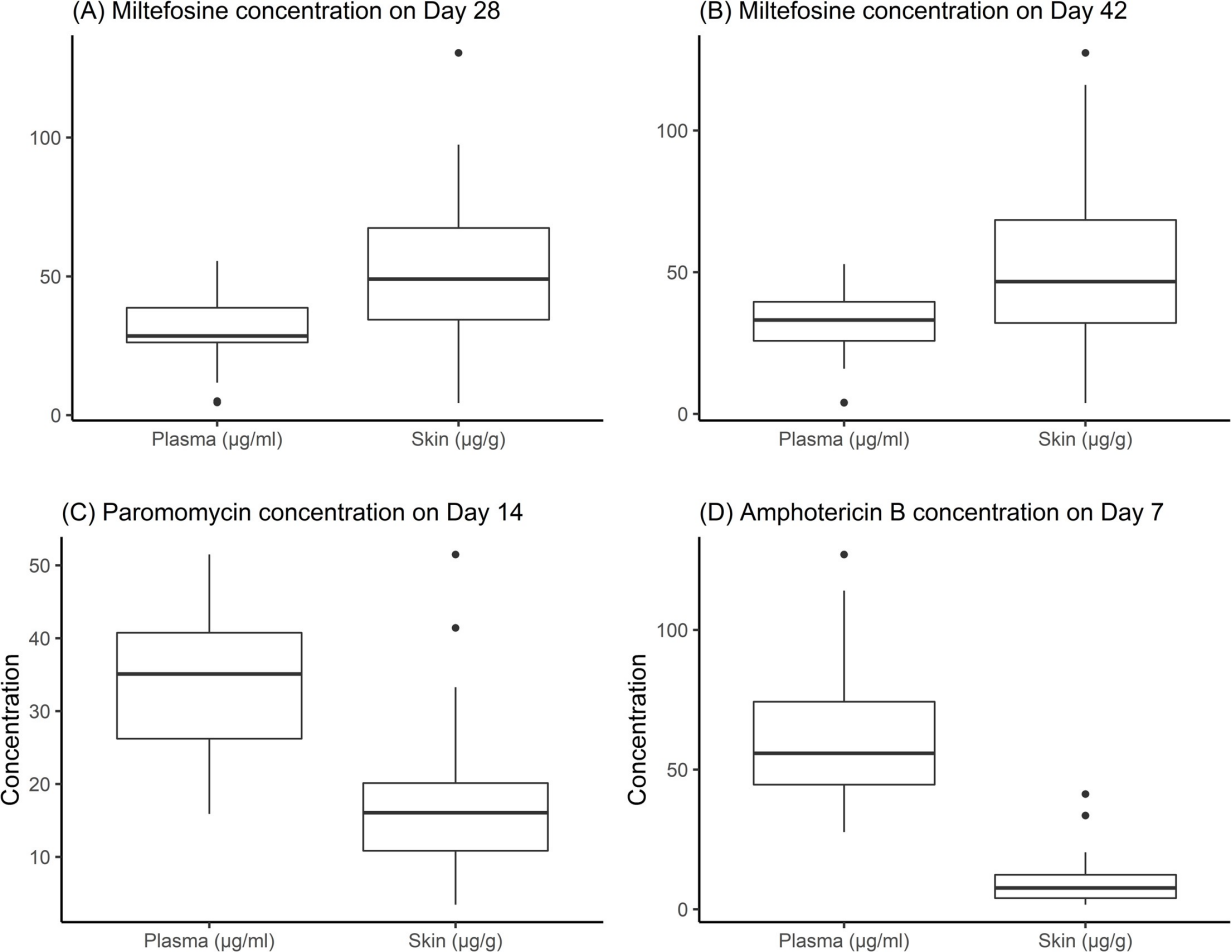
Median plasma and skin concentrations of miltefosine on days 28 and 42, of paromomycin on day 14, and liposomal amphotericin B on day 7. (A) Median plasma and skin concentration of miltefosine on day 28 (arm 2) were 28.5 μg/ml and 49 μg/g, respectively, with median skin to plasma concentration ratio calculated at 1.64 (IQR = 0.62–4.35); (B) Median plasma and skin concentration of miltefosine on day 42 (arm 1) were 32.9 μg/ml and 46.7 μg/g, respectively, with median skin to plasma concentration ratio calculated at 1.46 (IQR = 0.57–3.23); (C) Median plasma and skin concentration of paromomycin on day 14 (arm 1, intensive PK cohort) were 40.5 μg/ml and 17.3 μg/g, respectively, with median skin to plasma concentration ratio calculated at 0.44 (IQR = 0.2–1.05); (D) Median plasma and skin concentration of LAmB on day 7 (arm 2) were 55.8 μg/ml and 7.63 μg/g, respectively, with median skin to plasma concentration ratio calculated at 0.11 (IQR = 0.02–0.94).

MF concentrations in both plasma and skin reached a steady state by day 28, which continued to day 42 for arm 1, with a median skin to plasma concentration ratio of 1.46. Fast plasma clearance of PM was observed on the first and last days (days 1 and 14) of treatment in the intensive PK cohort, with maximum plasma values reached 1 hour after treatment (arm 1). PM plasma exposure was higher on day 14 compared to day 1, suggesting time-varying drug clearance. The median skin to plasma concentration ratio of PM was 0.44, which was calculated based on patients with plasma samples collected between 0.5 and 2.5 hours after the day 14 dose administration (around C_max_). Higher plasma LAmB exposure was observed on the last day of treatment (day 7) compared to day 1 in the intensive PK cohort, indicating potential non-linear kinetics of this drug (arm 2). At day 7, LAmB concentrations in the skin were considerably lower than in plasma, with a median skin to plasma concentration ratio of 0.11. This implies a limited extent of biodistribution to skin tissue, potentially leading to relatively low drug accumulation in skin by day 7. Available plasma and skin concentrations of MF, PM, and LAmB are depicted in Fig 4. More details of the PK and full description of pharmacodynamics data, based on quantitative parasite load in skin, will be presented in a separate manuscript. Overall, a significant decrease in parasite load was observed for the 2 study arms (p <0.0001), already at the Day 42 (EOT) assessment and maintained during the follow-up period.

## Discussion

This is the first randomized clinical trial to assess safety and efficacy of treatments for patients with PKDL in Sudan. The trial showed that MF combined with either PM or LAmB achieved clinically meaningful rates of definitive cure at 12 months follow-up in paediatric and adult patients with PKDL in Sudan. No new safety signals were reported and both combinations had favourable risk/ benefit profiles, with no reports of SAEs or deaths during the study. Outpatient treatment with MF was well tolerated overall, with good rates of treatment adherence.

The PM/MF combination achieved a 100% cure rate at 12 months in the complete case scenario, including patients with moderate and severe disease at baseline, and was well tolerated; the large majority of ADRs were mild episodes of vomiting, and only one patient had treatment discontinued due to AE. The LAmB/MF combination was also efficacious, with a cure rate of 86% in the complete case analysis. Cases of failure were mainly patients presenting with moderate/severe PKDL at baseline, who relapsed during the 12 months of follow-up, one case of no response to therapy, and one case of hypersensitivity to LAmB. All these patients received rescue therapy. This difference in efficacy may be explained by the different skin exposures for PM and LAmB, with skin to plasma concentration ratios of 0.44 and 0.1, respectively, and the longer MF regimen in PM/MF arm.

Despite the challenges of implementing this trial during the COVID-19 pandemic, only 3 patients were lost to follow-up (2.7%), and 2 withdrew consent (1.8%) during the trial. Furthermore, only 5 patients in Arm 1 and 4 in Arm 2 were not included in the PP population (due to poor compliance, exclusion criteria not met, or consent withdrawal), yielding similar efficacy rates in the mITT, PP and the complete case analyses.

A *post-hoc* analysis of potential prognostic factors for treatment outcome demonstrated a statistically significant 10-fold increase in risk of failure in patients with grade 3 PKDL lesions at baseline, but no other statistically significant risk factors were identified. The study was not powered for this analysis, limiting the conclusions to be drawn.

Consistent with epidemiological studies of VL in Sudan [35], the majority of patients in this study were children and male, with a median age (9 years) that was slightly higher than the most common age range reported for PKDL in Sudan [1]. The requirement for a pregnancy test and contraception during treatment and for 5 months afterwards (due to the teratogenic potential of MF) may explain the very low number of adolescent and adult female patients participating in the study. Patients had long history of PKDL (median time of 18 months) at study entry, which may have represented a potential source of infection for the communities. The majority of patients presented with maculopapular or papular PKDL lesions, in line with the most common PKDL presentation in Sudan [36].

Very few studies have been conducted in patients with PKDL in East Africa. An observational study in India based on 32 mostly adult patients with predominantly nodular, papular, or macular PKDL lesions showed a 75% (12/16) cure rate with MF alone (100 mg/kg/day for 90 days) and a 100% (16/16) cure rate with LAmB/MF combination (LAmB given in 3 doses of 5 mg/kg IV over 2 weeks and MF at 100 mg/kg/day for 45 days) [20]. However, our study was conducted in a different population, with a different LAmB /MF regimen, and a larger sample size. In Sudan, only small studies in patients with persistent PKDL have been conducted, with cure rates of 83% (10/12) with LAmB alone (2.5 mg/kg/day for 20 days) [11], 89% (8/9) with a combination of SSG and PM (20 mg/kg/day and 15 mg/kg/day, respectively, for 30 to 40 days) [12], and of 40% (6/15) with SSG (20 mg/kg/day for 40 days) + placebo at 90 days follow-up [37]. Our study showed better efficacy results with the combination of PM/MF, with a larger sample size, in patients with grade 1, 2, or 3 PKDL. One limitation of PKDL studies in Sudan is the potential progression to self-healing. In the present study, only patients with stable PKDL or worsening disease were included, which limits this risk, but this effect cannot be completely discarded in the efficacy estimations of this or any other trial.

The safety findings showed that these combination therapies are suitably safe for patients with PKDL. No SAEs or deaths were reported, and the vast majority of AEs were of mild or moderate intensity. Vomiting was the most frequent AE, as in previous studies with miltefosine [38], and occurred mainly during the first 2 weeks of therapy; the higher frequency of vomiting in arm 2 was mainly observed in the first week of therapy and could not be explained by exposure to miltefosine. Only two patients discontinued treatment: one patient in the LAmB/MF arm, due to well-described hypersensitivity to LAmB (on Day 1, at first administration, requiring rescue therapy), and another patient in the PM/MF arm who presented acute kidney injury (creatinine levels increased from grade 1 to grade 2 CTCAE grading) and whose MF treatment was discontinued on Day 21. Considering the significant improvement in the PKDL lesions at time of treatment discontinuation, the investigator did not indicate rescue therapy and patient evolved with cure. Hypokalaemia related to LAmB therapy was reported in 12.7% of the patients in LAmB/MF arm, similar to that reported for LAmB treatment of PKDL in Asia [19]. No cases of rhabdomyolysis were observed, as have been described in Bangladesh for patients treated with a total LAmB dose of 30mg/Kg [39]. Unlike patients with VL who have been treated with same regimen of PM [3], no hearing impairment was observed in patients with PKDL. A more detailed comparison of pharmacokinetic characteristics between these two patient populations may bring some insight into this difference, which might also be explained by the overall condition of patients with PKDL, with better nutritional status and less risk of other co-morbidities. Finally, recently there has been a lot of attention given to the risk of ocular toxicity, mainly associated with the 12-week MF therapy for PKDL in South Asia [40–42]. In Sudan, post-kala-azar ocular leishmaniasis has been described by Prof. EL-Hassan *et al*. concomitantly with PKDL [36], with presentations of conjunctivitis and uveitis. In this study, none of the patients had ocular findings at admission, and only one case of conjunctivitis considered not related to the study drug was reported, treated, and resolved.

The current standard-of-care for treatment of PKDL in Sudan, SSG [13], is not suitable for use in otherwise well patients because of its toxicity and the need for lengthy hospitalization. Based on our efficacy and safety findings, PM/MF seems to be a suitable alternative option for treatment of patients with PKDL in Sudan, even for patients with moderate and severe PKDL. This regimen is of shorter duration than current options, and can be administered at primary health care level. This more patient-friendly treatment is particularly valuable since the majority of PKDL patients are children. For those patients unable to tolerate PM due to renal or hearing issues, the LAmB/MF combination is a good alternative, however, it must be administered in higher complexity settings since serum potassium and hepatic function need to be closely monitored.

For patients who cannot be treated with MF or women of childbearing potential who are not willing to use contraception, the SSG/PM combination can be an alternative. Pregnant women could either delay the therapy for PKDL or receive treatment with LAmB. After adoption of these therapies, effectiveness studies will be needed to confirm these results in field conditions.

Patients with PKDL in Sudan present the clinical manifestations soon after being cured of VL, the majority within 6 to 12 months of VL therapy; most patients present papular or maculo-papular lesions [1]. Self-healing is also reported, which is why only patients with persistent or progressive disease were enrolled in this trial. Response to therapy was observed rapidly, with approximately half of patients completely healed by Day 42 and the majority cured by Day 90. This differs from patients with PKDL in Asia, where most present macular lesions that take much longer to re-pigment. In routine care in Sudan, as per local guidelines, resolution of PKDL is assessed based on clinical presentation, there is no parasitic criteria for cure. The scoring system used in this trial, despite being semi-quantitative, proved to be a useful measurement of the healing process. This rapid response to therapy in Sudan may provide an opportunity for assessing future new therapies for PKDL, where proof of concept trials or dose-finding studies can be designed with early efficacy endpoints. Such studies could use a composite endpoint, combining clinical and parasitological and/or immunological endpoints, which might provide insight into the possibility of a considerably shorter duration of treatment [43]. Nevertheless, in definitive trials, long-term follow-up would still be needed to assess the risk of late relapse, as observed with LAmB/MF therapy in this study.

While the positive results of this trial represent a significant improvement on existing options and could reduce VL transmission and hence help in disease control, efficacious, safe, oral treatments need to be developed for all patients with PKDL in Eastern Africa, including mild cases and women of childbearing potential unwilling or unable to use contraception. Community engagement and educational programs are needed to raise awareness of the disease and the availability of treatments, to reduce time from PKDL onset and access to diagnosis and treatment. Finally, better treatments are needed for VL that have a reduced risk of subsequent development of PKDL, comprising anti-parasitic drugs and, potentially, host-directed immune-modulatory therapy. Only then can transmission of VL be sufficiently reduced to reach the WHO aim of eliminating the disease as a public health problem, as highlighted in the WHO NTD Roadmap [18].

## Supporting information

S1 File Supplementary material. (PDF)

S1 Trial Protocol PKDL Sudan Study Synopsis. (PDF)

S1 CONSORT Checklist CONSORT 2010 checklist of information to include when reporting a randomised trial. (PDF)
