# Supplementary material for: Safety and efficacy of paromomycin/miltefosine/liposomal amphotericin B combinations for the treatment of post-kala-azar dermal leishmaniasis in Sudan: A phase II, open label, randomized, parallel arm study
Source: PLoS Negl Trop Dis. 2023 Nov 21;17(11):e0011780. doi: 10.1371/journal.pntd.0011780 (PMC10721181; doi:10.1371/journal.pntd.0011780)
Supplement: S1 File — (PDF) [file pntd.0011780.s001.pdf]

Supplementary material

Text A. Clinical Assessments of Lesions – grading of PKDL

Clinical evolution was recorded systematically by the Investigator during the screening period (Days -30 to 0), at Day 42 (EOT), and at the follow-up visits at 3, 6, and 12 months after treatment onset.

At each of these visits, the PKDL lesions were assessed for grading, describing both the distribution and the density of the lesions, as follows:

- Distribution of the lesions:
    - Grade 1: face mainly with some lesions on the trunk and arms;
    - Grade 2: face, upper parts of the trunk, arms, and legs affected, gradually becoming less distal; hand and feet free of lesions;
    - Grade 3: all over the body, including the hands and feet.
- Density of the lesions:
    - Grade 1: scattered lesions;
    - Grade 2: moderate density with normal skin in between lesions;
    - Grade 3: dense rash, no normal skin.

Table A. Schedule of assessments

Protocol activities	Screening	Treatment period					EOT	Follow-up period		
	D-30 to D0	D1	D3	D7	D14	D28	D42	3M	6M	12M
Consent form	X									
Inclusion and exclusion criteria	X									
Demographic data and medical history	X									
Vital signs and physical exam	X	X	X	X	X	X	X	X	X	X
Audiometric test*	X						X[a]		X[a]	X[a,b]
HIV test	X									
Pregnancy test [c]	X						X	X	X	X[a]
Depo-Provera® injection [c]	X							X	X[a]	
Haematology Hb, RBC, WBC, platelets	X		X	X	X	X	X	X[b]	X	X
Chemistry Albumin**, ALT, AST, bilirubin, creatinine, potassium	X	X	X	X	X	X	X	X[b]	X[b]	X[b]
Skin biopsy for PK				X[d]	X[a]	X[d]	X[a]			
Blood sample for PK										
PK amphotericin B		X[e]		X[f]						
PK PM		X[e]			X[f]					
PK MF		X		X	X	X	X	X		
PKDL evolution assessment, including photographs***	X						X	X	X	X
Safety assessment	SAEs	------- SAEs and AEs monitoring -------						S/AEs[g]		
Study treatment		------------ AmB + MF -----------								
		------------------- PM + MF -----------------								

AE = Adverse event; ALT = Alanine aminotransferase; AmB = liposomal amphotericin B; AST = Aspartate aminotransferase; D = Day; EOT = End of treatment; Hb = Hemoglobin; HIV = Human immunodeficiency virus; M = Month; MF = Miltefosine; PK = Pharmacokinetic(s); PKDL = Post-kala-azar dermal leishmaniasis; PM = Paromomycin; RBC = Red blood cell; SAE = Serious adverse event; WBC = White blood cell.

a) Patients allocated to Arm 1 (PM/MF).
b) Only if abnormal at the previous assessment in a scheduled or unscheduled visit.
c) For women of child-bearing potential only. First injection to be given after consent and eligibility for the study has been confirmed.
d) Patients allocated to Arm 2 (AmB/MF).
e) Multiple sampling for the subset of 30 patients only. No sampling for other patients.
f) Multiple sampling for a subset of 30 patients only, single sampling for other patients.
g) Between 6 and 12 months follow-up, all SAEs (related or not) and only non-serious AEs related to study drug were collected.
* As per study SOP
** Albumin was measured at screening only.
*** PKDL evolution assessment and photographs could be done at any time if rescue treatment was indicated.

Table B. Baseline laboratory parameters by treatment arm and overall – ITT set

Parameter	Statistics	Arm 1 PM/MF (n=55)	Arm 2 LAmB/MF (n=55)	Overall (n=110)
Haemoglobin (g/dL)	Range (Min; Max)	8.7; 16.3	8.8; 15.4	8.7; 16.3
	Mean (SD)	12.5 (1.3)	12.3 (1.2)	12.4 (1.3)
	Median (IQR)	12.5 (11.8; 13.2)	12.2 (11.7; 13.2)	12.3 (11.7; 13.2)
WBCs (x10^3/µL)	Range (Min; Max)	3.7; 37.9[1]	3.1; 10.7	3.1; 37.9[1]
	Mean (SD)	7.5 (4.5)	6.8 (1.6)	7.2 (3.4)
	Median (IQR)	6.5 (5.9; 8.2)	6.7 (5.7; 8.1)	6.5 (5.8; 8.2)
Platelets (x10^3/µL)	Range (Min; Max)	170.0; 830.0	167.0; 605.0	167.0; 830.0
	Mean (SD)	351.3 (105.0)	331.7 (84.5)	341.5 (95.4)
	Median (IQR)	341.0 (294.0; 399.0)	325.0 (266.0; 390.0)	338.0 (284.0; 391.0)
ALT (U/L)	Range (Min; Max)	10.0; 90.0	9.0; 60.0	9.0; 90.0
	Mean (SD)	26.8 (14.7)	26.3 (9.6)	26.5 (12.4)
	Median (IQR)	23.0 (17.0; 31.0)	24.0 (20.0; 31.0)	24.0 (19.0; 31.0)
AST (U/L)	Range (Min; Max)	10.0; 67.0	18.0; 56.0	10.0; 67.0
	Mean (SD)	30.5 (10.8)	31.7 (9.2)	31.1 (10.0)
	Median (IQR)	29.0 (24.0; 36.0)	30.0 (25.0; 36.0)	29.5 (24.0; 36.0)
Total bilirubin (mg/dL)	Range (Min; Max)	0.1; 1.5	0.2; 1.1	0.1; 1.5
	Mean (SD)	0.5 (0.3)	0.4 (0.3)	0.5 (0.3)
	Median (IQR)	0.4 (0.3; 0.7)	0.4 (0.2; 0.7)	0.4 (0.2; 0.7)
Creatinine (mg/dL)	Range (Min; Max)	0.1; 1.0	0.1; 0.8	0.1; 1.0
	Mean (SD)	0.4 (0.2)	0.4 (0.2)	0.4 (0.2)
	Median (IQR)	0.4 (0.3; 0.5)	0.4 (0.2; 0.6)	0.4 (0.2; 0.5)
Potassium (mmol/L)	Range (Min; Max)	3.1; 4.8	3.3; 4.8	3.1; 4.8
	Mean (SD)	4.0 (0.3)	4.0 (0.3)	4.0 (0.3)
	Median (IQR)	4.0 (3.7; 4.2)	4.0 (3.8; 4.3)	4.0 (3.8; 4.2)

ALT = Alanine aminotransferase; LAmB = Liposomal amphotericin B; AST = Aspartate aminotransferase; MF = Miltefosine; PM = Paromomycin

Table C. Primary efficacy outcome of definitive cure at 12 months by treatment arm – complete case analysis in mITT and PP sets

		mITT		PP	
	Statistics	Arm 1 PM/MF	Arm 2 LAmB/MF	Arm 1 PM/MF	Arm 2 LAmB/MF
**Complete case scenario**[1]	n	54	51	49	49
	Number cured	54	44	49	42
	Efficacy, %	100.0	86.3	100.0	85.7
	95% CI	(93.4; 100.0)	(73.7; 94.3)	(92.7; 100.0)	(72.8; 94.1)

LAmB = liposomal amphotericin B; CI = Confidence interval; MF = Miltefosine; mITT = Modified intention-to-treat; PM = Paromomycin; PP = Per-protocol.
[1] Complete case scenario (sensitivity analysis): missing efficacy outcome at 12 months excluded from the analysis.

Table D. Post-hoc analysis of the odds of failure by baseline PKDL grade, initial disease status, duration of PKDL onset, age, and sex – mITT set

	Odds ratio	95% CI	p-value
**PKDL grade** (reference: Grade 1)			
Grade 2	2.95	0.5; 18.6	0.249
Grade 3	10.17	1.2; 85.7	0.033
**Duration of PKDL onset** (reference: <1 year)			
1-2 years	3.81	0.4; 38.8	0.259
>2 years	2.54	0.3; 25.6	0.429
**Initial disease status** (reference: stable)			
Worsening	4.11	0.9; 19.7	0.077
**Age group** (reference: <10 years)			
10-30 years	0.69	0.1; 3.7	0.666
**Sex** (reference: male)			
Female	0.45	0.1; 2.1	0.320

CI = Confidence interval; mITT = Modified intention-to-treat; PKDL = Post-kala-azar dermal leishmaniasis.
Note: A univariate logistic regression analysis was used and includes all patients from both treatment arms.

Fig A. Mean profile plot for skin lesions score by treatment arm over time – mITT set

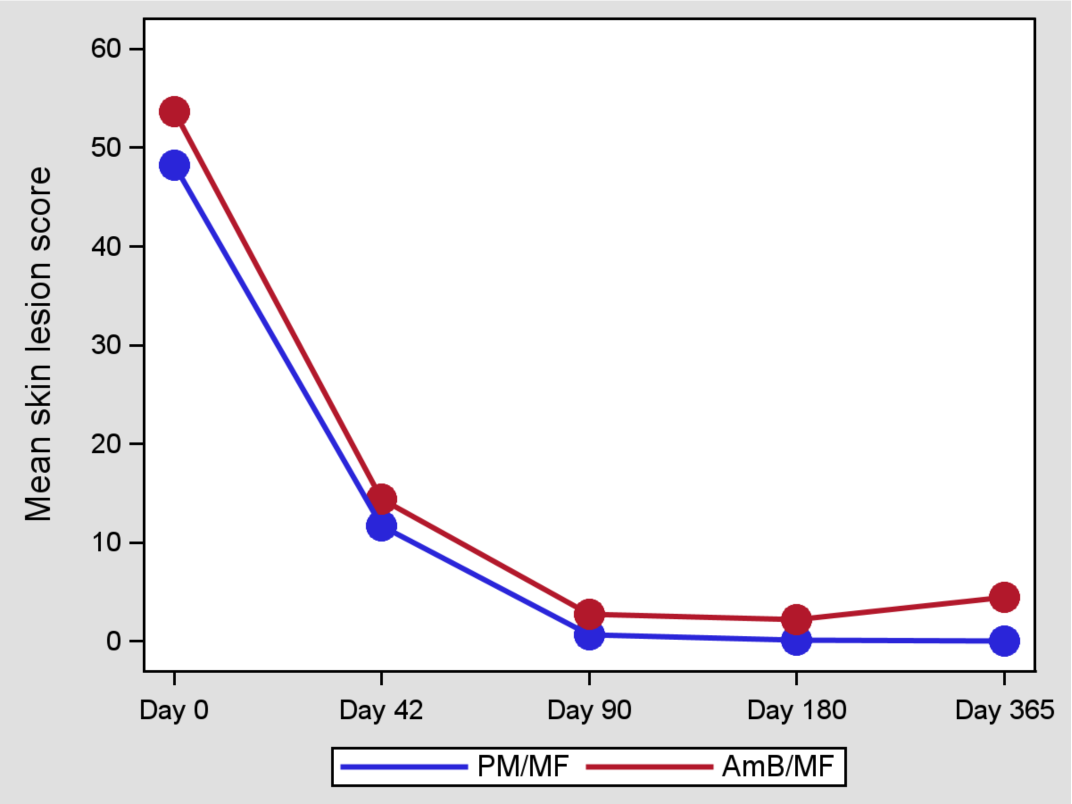

AmB = liposomal amphotericin B; MF = Miltefosine; PM = Paromomycin.

Fig B. PKDL lesion density grade over time by treatment arm – mITT set

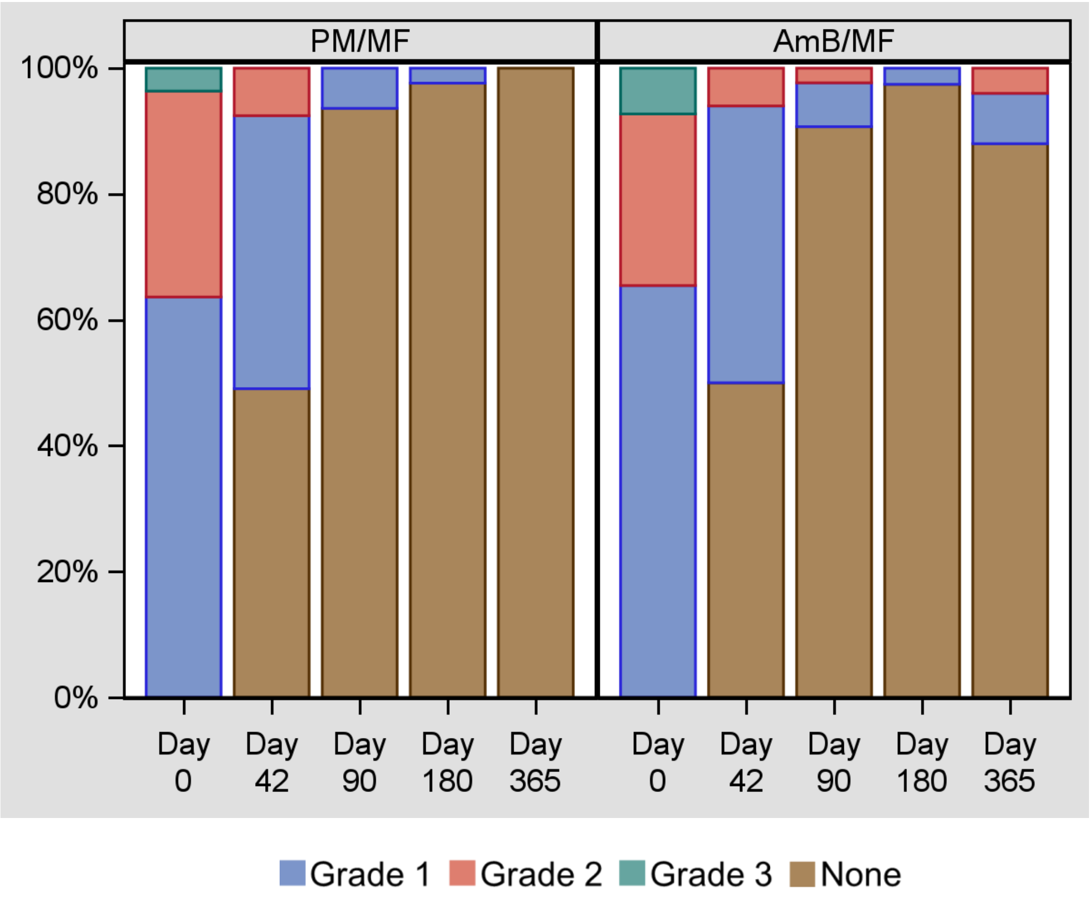

AmB = liposomal amphotericin B; MF = Miltefosine; mITT = Modified intention-to-treat; PKDL = Post-kala-azar dermal leishmaniasis; PM = Paromomycin.
Note: The grade "none" refers to the absence of lesions in the patients, i.e., the patients are cured.

Fig C. Kaplan-Meier curve of time to treatment failure by treatment arm and overall – mITT set

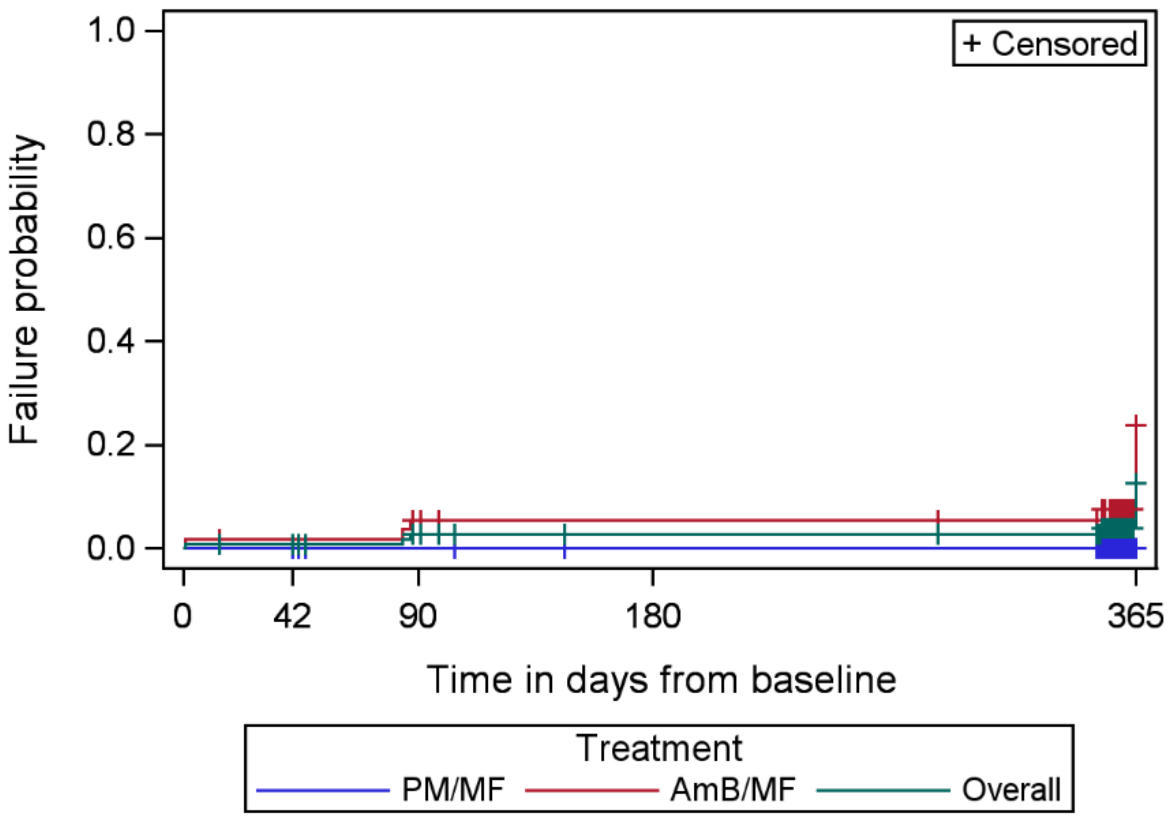

LAmB = liposomal amphotericin B; MF = Miltefosine; PM = Paromomycin.
