# Supplementary material for: Safety and efficacy of paromomycin/miltefosine/liposomal amphotericin B combinations for the treatment of post-kala-azar dermal leishmaniasis in Sudan: A phase II, open label, randomized, parallel arm study
Source: PLoS Negl Trop Dis. 2023 Nov 21;17(11):e0011780. doi: 10.1371/journal.pntd.0011780 (PMC10721181; doi:10.1371/journal.pntd.0011780)
Supplement: S1 Trial Protocol — (PDF) [file pntd.0011780.s002.pdf]

PKDL Sudan Study Synopsis

**Protocol Title**	An Open label, Randomized, Parallel arm Clinical Trial of Two Regimens to Assess the Safety and Efficacy for Treatment of Post Kala-azar Dermal Leishmaniasis (PKDL) Patients in Sudan
**Phase**	Phase II
**Indication**	PKDL patients in Sudan
**Protocol Number**	DNDi-MILT COMB-02-PKDL
**Trial Objectives**	General Objectives:<br>The overall objective of this study is to assess the safety and efficacy of two treatment modalities for PKDL patients in Sudan.<br>Primary Objective:<br>To assess the safety and efficacy of Paromomycin combined with Miltefosine and AmBisome [redacted] combined with Miltefosine for the treatment of PKDL in Sudan.<br>Secondary Objectives:<br>• To assess skin and plasma concentrations of Paromomycin, amphotericin B and Miltefosine and how drug exposure in skin and plasma relates to the in vitro susceptibility of the causative *Leishmania* species for each compound.<br>• To evaluate the host immune response in each treatment arm before, during and after treatment.<br>• To evaluate parasite clearance in each arm as indicated by direct microscopy and qPCR<br>• To compare clinical, pharmacological, parasitological and immunological responses to identify a potential biomarker for cure<br>• To assess relationship between pharmacokinetic parameters with clinical outcome and parasite clearance.<br>Exploratory objectives:<br>• To assess non-invasive tape disc method of skin samples collection for molecular parasitological diagnosis.
**Trial Endpoints**	Primary Endpoints:<br>• **Efficacy**<br>The primary endpoint variable is definitive cure at 12 months after

	treatment onset, defined as clinical cure (100% lesions resolution) and no additional PKDL treatment between end of therapy and 12 months follow-up assessment.<br>• **Safety**<br>o Serious adverse events from the start of treatment through 12-month follow period<br>o Frequency and severity of adverse events that lead to treatment discontinuation.<br>o Frequency and severity of all adverse events from the start of treatment through 12-month follow up period<br><br><u>Secondary Endpoints</u>:<br>**Pharmacokinetics**<br>To assess the maximal accumulation of Paromomycin, total amphotericin B and Miltefosine in the skin at the end of treatment and correlate these with achieved plasma concentrations.<br>**Immune Response**<br>To assess the change in immune response during and after end of treatment by measuring cytokines profiles level in the peripheral blood.<br>**Parasitology**<br>To assess the clearance of parasites by microscopy and qPCR in blood and skin, at various time-points during and after treatment and during follow-up.
**Trial Design**	This is an open label, randomized non comparative phase II clinical trial conducted on parallel groups, to assess the safety and efficacy of the combination of Paromomycin (20 mg/kg/d) IM for 14 days and Miltefosine (allometric dosing) oral for 42 days, and a combination of AmBisome[redacted] (20 mg/kg total dose) IV over 7 days and Miltefosine oral for 28 days (allometric dosing) for the treatment of PKDL patients in Sudan.<br>Evaluation of the response to treatment will be done by clinical assessment which includes the comparison of photographs taken under standardized conditions.<br>• <u>Improvement</u> is defined as 1) appearance: darkening of macular lesions and flattening of papular or nodular lesions, and/or 2) extension: reduction in extent of the rash.<br>• Definitive <u>Cure</u> at 12 months is defined as complete disappearance of all lesions.<br>• *Subjects will be hospitalized during the administration of AmBisome[redacted] or Paromomycin.*<br>• *The Miltefosine treatment will start at the same time as AmBisome[redacted] or Paromomycin treatment and will continued on an out-patient basis until completion of 28 days (MF/AmBisome[redacted]) or 42 days (MF/PM).*

	During hospitalization patients will be instructed on Miltefosine treatment, the daily dose (morning and afternoon), and the administration with food to avoid vomiting. Miltefosine treatment as out-patient will be under direct observed treatment (DOT) by the community health worker at the village level, whenever possible.<br>*After a screening period, the study visits will be at D1, D3, D7, D14 and D28. The end of treatment (EOT) assessment will be done at D42, followed by follow-up visits 3, 6 and 12 months after the onset of treatment to assess efficacy and safety.*<br>*The patients will be instructed to return to the clinic at any point in time during follow-up if they present any medical condition or if the PKDL lesions*
**Study Duration**	6 Months are expected to be needed to obtain all ethical and official clearances. The recruitment period will be 24 months and the follow-up period will be 12 months. Therefore, FPI to LPO will be 36 months.
**Test Drugs**	1. Paromomycin 20 mg/kg/d IM for 14 days combined with Miltefosine allometric BID PO dosing for 42 days<br>2. AmBisome[redacted] 5mg/kg/d IV infusion at D1, D3, D5 and D7 (20 mg/kg total dose) combined with Miltefosine allometric BID PO dosing for 28 days<br>For Miltefosine allometric dosing an easy-to-use table with dosing scheme by weight and height will be provided to the investigators to define the exact daily dose to be administered.
**Sample size**	55 subjects will be included in each arm. The overall sample size for the two regimens is 110 subjects.
